# Construction and analysis of tag single nucleotide polymorphism maps for six human-mouse orthologous candidate genes in type 1 diabetes

**DOI:** 10.1186/1471-2156-6-9

**Published:** 2005-02-18

**Authors:** Lisa M Maier, Deborah J Smyth, Adrian Vella, Felicity Payne, Jason D Cooper, Rebecca Pask, Christopher Lowe, John Hulme, Luc J Smink, Heather Fraser, Carolyn Moule, Kara M Hunter, Giselle Chamberlain, Neil Walker, Sarah Nutland, Dag E Undlien, Kjersti S Rønningen, Cristian Guja, Constantin Ionescu-Tîrgovişte, David A Savage, David P Strachan, Laurence B Peterson, John A Todd, Linda S Wicker, Rebecca C Twells

**Affiliations:** 1 Juvenile Diabetes Research Foundation/Wellcome Trust Diabetes and Inflammation Laboratory, Cambridge Institute for Medical Research, University of Cambridge, Wellcome Trust/MRC Building, Hills Road, Cambridge, UK; 2 Institute and Department of Medical Genetics, Ulleval University Hospital, University of Oslo, Oslo, Norway; 3 Laboratory of Molecular Epidemiology, Division of Epidemiology, Norwegian Institute of Public Health, Oslo, Norway; 4 Clinic of Diabetes, Institute of Diabetes, Nutrition and Metabolic Diseases 'N. Paulescu', Bucharest, Romania; 5 Department of Medical Genetics, Queen's University Belfast, Belfast City Hospital, Belfast, UK; 6 Department of Community Health Sciences, St George's Hospital Medical School, London, UK; 7 Department of Pharmacology, Merck Research Laboratories, Rahway, New Jersey, USA

## Abstract

**Background:**

One strategy to help identify susceptibility genes for complex, multifactorial diseases is to map disease loci in a representative animal model of the disorder. The nonobese diabetic (NOD) mouse is a model for human type 1 diabetes. Linkage and congenic strain analyses have identified several NOD mouse *Idd *(insulin dependent diabetes) loci, which have been mapped to small chromosome intervals, for which the orthologous regions in the human genome can be identified. Here, we have conducted re-sequencing and association analysis of six orthologous genes identified in NOD *Idd *loci: *NRAMP1/SLC11A1 *(orthologous to *Nramp1/Slc11a1 *in *Idd5.2*), *FRAP1 *(orthologous to *Frap1 *in *Idd9.2*), *4-1BB/CD137/TNFRSF9 *(orthologous to *4-1bb/Cd137/Tnrfrsf9 *in *Idd9.3*), *CD101/IGSF2 *(orthologous to *Cd101/Igsf2 *in *Idd10*), *B2M *(orthologous to *B2m *in *Idd13*) and *VAV3 *(orthologous to *Vav3 *in *Idd18*).

**Results:**

Re-sequencing of a total of 110 kb of DNA from 32 or 96 type 1 diabetes cases yielded 220 single nucleotide polymorphisms (SNPs). Sixty-five SNPs, including 54 informative tag SNPs, and a microsatellite were selected and genotyped in up to 1,632 type 1 diabetes families and 1,709 cases and 1,829 controls.

**Conclusion:**

None of the candidate regions showed evidence of association with type 1 diabetes (*P *values > 0.2), indicating that common variation in these key candidate genes does not play a major role in type 1 diabetes susceptibility in the European ancestry populations studied.

## Background

Type 1 diabetes is a common, multifactorial disease believed to be caused in a proportion of cases by an autoimmune destruction of pancreatic β-cells by an inflammatory infiltrate comprising T lymphocytes, dendritic cells and macrophages. This process results from a complex interaction between genetic and environmental risk factors. Genetically, it is under the control of the major histocompatibility complex (MHC) [1] and many other genes of smaller effect and mostly unknown identity.

A murine model of type 1 diabetes, the NOD mouse, spontaneously develops an autoimmune-mediated diabetes that has many similarities to the human disease. It is likely that components of the pathophysiology and genetic predisposition are conserved across species, and indeed two loci have already been shown to affect type 1 diabetes susceptibility in both species, namely the immunoregulatory MHC HLA class II and CTLA-4 genes. The other causative gene(s) in the known *Idd *regions controlling type 1 diabetes susceptibility in the NOD mouse could also determine susceptibility in humans, even though this depends on the frequency of susceptibility alleles in human populations, which affects statistical power, and that the correct candidate gene has been chosen from the *Idd *interval. These *Idd *intervals might contain many genes, including several involved in the immune response [2]. Nevertheless, in contrast to studies in humans based on linkage, the localisation of a type 1 diabetes locus to a specific chromosome region in the mouse genome using congenic strain breeding defines with certainty a set of genes, one or more of which is definitely a susceptibility gene [3,4].

The central importance of T cell development and function in type 1 diabetes is evident from the susceptibility genes identified so far. The MHC class II genes are important etiologically in two rat models of type 1 diabetes, the Biobreeding (BB) and KDP strains [5,6], the NOD mouse strain [3] and in humans [1], with their essential function not only in T cell activation and expansion but also in T cell repertoire formation in the thymus and clonal deletion of autoreactive cells. The BB rat type 1 diabetes susceptibility locus *Ian4/Iddm1 *[7] affects T lymphocyte development whereas the *Cblb *(KDP rat) [8] and *CTLA4 *[9] (in humans and NOD mice) susceptibility genes highlight the importance of the regulation of T cell activation, expansion and homeostasis in the periphery, and perhaps in the thymus as well.

In our selection of candidate genes within NOD congenic intervals, we have, therefore, biased our choice towards immune-related genes such as *Il2 *[2], *Cd101 *[10] and *Nramp1/Slc11a1 *[11]. From each of *Idd5.2 *[11], *Idd9.2 *[12], *Idd9.3 *[12], *Idd10 *[10], *Idd13 *[13] and *Idd18 *[14] we chose immune-associated functional candidate genes to study in human type 1 diabetes: *Nramp1*/*Slc11a1 *from *Idd5.2 *[11]; *Frap1 *from *Idd9.2 *(unpublished); *4-1bb/Cd137/Tnfrsf9 *from *Idd9.3 *[12] (unpublished); *Cd101/Igsf2 *from *Idd10 *[10]; *B2m *from *Idd13 *[15] and *Vav3 *from *Idd18 *(note that very recent congenic strain mapping results indicate that the *Idd18 *interval contains only one gene with known immunological function, namely the VAV3 gene, and this will be published elsewhere). Table 1 summarises the main features of the six human candidate genes.

**Table 1:** NOD mouse *Idd *loci, location of their human orthologous regions, and selected functional candidate genes.

***Idd***	**Mouse chromosome**	**Interval size (Mb)**	**Number of genes**	**Functional candidate genes in mouse *Idd *intervals**	**Location of human orthologous region**	**Human orthologue genes**	**Known gene function and previously reported disease associations**
*Idd5.2*	1	1.7	47	*Idd5.2: Nramp1/Slc11a1*	2q35	*NRAMP1/SLC11A1*	Endosomal/lysosomal acidification and associated with protection from infectious disease and susceptibility to autoimmune disease
*Idd9.2*	4	1.1	13	*Idd9.2: Frap1*	1p32	*FRAP1*	FKBP12-rapamycin associated protein of mTOR. Candidate tumour suppressor gene, whose function in apoptosis is influenced by allelic variation
*Idd9.3*	4	1.2	13	*Idd9.3: 4-1bb*	1p36	*4-1BB*	Role in enhancing and regulating CD4^+^, CD8^+ ^T cells and dendritic cells
*Idd10*	3	0.95	7	*Idd10: Cd101*	1p12	*CD101*	Co-stimulatory receptor of T cells
*Idd13*	2	6 cM	> 50*	*Idd13: B2m*	15q21	*B2M*	Required for antigen presentation by MHC class I molecules and the development of diabetes in NOD mice
*Idd18*	3	0.7	2	*Idd18: Vav3*	1p13-p21	*VAV3*	Guanine nucleotide exchange factor involved in signalling of T and B cell receptors

*Estimated number of genes. A version of this table is provided in Additional file 1 with the supporting published references (see Additional file 1).

## Results and discussion

A tag SNP approach to test for association was adopted for all genes, except for *4-1BB *[16], in order to achieve cost-savings in genotyping. A multi-locus test was used to evaluate the association between type 1 diabetes and the tag SNPs due to linkage disequilibrium (LD) with one or more causal variants [17]. Coding and untranslated regions of *NRAMP1 *(MIM 600266), *FRAP1 *(MIM 601231), *4-1BB *(MIM 602250), *CD101 *(MIM 604516), *B2M *(MIM 109700) and *VAV3 *(MIM 605541) were re-sequenced in 32 or 96 randomly chosen UK white patients with type 1 diabetes to identify SNPs and for the selection of tag SNPs. As LD between *4-1BB *SNPs was weak, eight out of nine common SNPs were genotyped (minor allele frequency, MAF ≥ 0.03; one SNP could not be genotyped due to assay technical difficulties) and analysed using single-locus tests.

A total of 110 kb of re-sequenced regions yielded 220 SNPs, including six deletion/insertion polymorphisms (DIPs) (see Table 2 and Additional files 2, 3,4,5,6 and7). No coding changes or obvious candidates for variants that could change the function or expression of *4-1BB, FRAP1*, or *B2M *were observed. A synonymous change was detected in exon 3 of *NRAMP1 *(MAF = 0.32) and a non-synonymous SNP (nsSNP) in exon 15 (MAF = 0.02), causing a conservative amino acid change: Asp543Asn (DIL5202/ss23142243). Interestingly, as in the case of its mouse orthologue [10], several nsSNPs were discovered in exons 3, 4, 5, and 8 of *CD101 *(see Additional file 5). Re-sequencing of the three alternative transcripts of *VAV3*, called *VAV3 *(27 exons), *VAV3β *(unique exon 1 and exons 4 to 27) and *VAV3.1 *(unique exon 18 and exons 19 to 27) yielded six exonic SNPs (see Additional file 7). Two SNPs, Pro611Ser (MAF = 0.13) and Gln613His (MAF = 0.13) are located in the SH3 domain of the VAV3 protein and, therefore, could result in VAV3 having altered protein interactions. In order to facilitate the computation of the selection of tag SNPs, *VAV3 *was divided into three sections as suggested by the pattern of LD across the gene.

**Table 2:** Summary of the re-sequencing study. Gene size, number of exons, amount of re-sequenced DNA for each gene (including 5' and 3' regions of gene), sequencing panel, and number of SNPs identified.

**Locus**	**Genomic size (kb)**	**n exons**	**Re-sequenced region (kb)**	**n cases re-sequenced**	**n SNPs**
*NRAMP1*	13.58	15 (7,13)*	12.13	32	20
*4-1BB*	20.50	8	13.66	96	23
*FRAP1*	60.98	58	30.88	32	55
*CD101*	34.61	10	15.90	96	31
*B2M*	6.61	4	9.33	32	13
*VAV3*	393.70	27 (25,10)*	27.69	96	78
*Total*	*529.98*	*122*	*109.59*	*-*	*220*

* Number of exons in splice variants. n, number.

Two common nsSNPs (MAF ≥ 0.05; DIL1521/rs7528153 and DIL3809/ss23142432) from *VAV3 *and a microsatellite from *NRAMP1 *were genotyped *a priori *in the whole family collection (step 1 and 2) and a single nsSNP from *CD101 *in step 1 families only (DIL3794/rs3754112). The nsSNP DIL3810/ss23142433 in *VAV3 *was not tested because it was in quite strong LD with DIL3809/ss23142432 (R^2 ^= 0.64), so that only DIL3809/ss23142432 was genotyped. Note that in our tag approach, the two VAV3 nsSNPs (DIL1521/rs7528153 and DIL3809/ss23142432) were chosen deliberately as tag SNPs.

In a pragmatic, phased genotyping strategy, in step 1, the multi-locus test *P *values for association between type 1 diabetes and candidate gene tag SNPs all exceeded 0.2, as did the single-locus test *P *values for *4-1BB *SNPs. Consequently, we did not proceed to genotype in step 2 samples for any of the candidate genes (Table 3 and 4). Note that none of the nsSNPs of *VAV3 *and *CD101 *or the microsatellite of *NRAMP1 *showed evidence of association (Table 5). Allele A3 of the *NRAMP1 *microsatellite promoter (GT)_n _has previously shown linkage and association with autoimmune disease, and allele A2 with infectious disease susceptibility [18-20]. The relative risks of allele A3 and genotype A3/A3 in our type 1 diabetes samples was 0.96 (95% CI = 0.94 – 1.17) and 0.90 (95% CI = 0.70 – 1.16), respectively.

**Table 3:** Study design. Lengths of re-sequenced genomic regions, and number of tag SNPs or single SNPs genotyped in a pragmatic two-step genotyping design for *NRAMP1*, *4-1BB*, *FRAP1*, *CD101*, *B2M*, and *VAV3*.

**Locus**	**Re-sequenced region (kb)**	n common SNPs*	**n tag SNPs**	**Genotyping strategy (step 1 → step 2)**
*NRAMP1*	12.13	12	4	Case-control → Family set 1+2
*4-1BB*	13.66	8	DIL4279/ss23142250	Family set 1
			DIL4277/rs226476	
			DIL4569/rs226478	
			DIL4274/ss23142263	
			DIL4570/ss23142264	
			DIL4571/rs679563	
			DIL4273/ss23142265	
			DIL4272/ss23142270	
*FRAP1*	30.88	21	6	Family set 1
*CD101*	15.9	18	8	Family set 1
*B2M*	9.33	10	8	Case-control → Family set 1
*VAV3*	27.69	19 (block 1) 18 (block 2) 15 (block 3)	7 (block 1) 11 (block 2) 10 (block 3)	Family set 1

*For the selection of tag SNPs, minor allele frequencies of 0.03 were used for *4-1BB*, *CD101 *and *FRAP1*, and 0.05 for *NRAMP1*, *B2M *and *VAV3*. Note that the numbers of attempted and actual genotypes are given in Additional file 8. n, number.

**Table 4:** Disease association results. Multi-locus test *P *values, lengths of re-sequenced genomic regions, and number of tag SNPs or single SNPs genotyped in a two-step genotyping design for *NRAMP1*, *4-1BB*, *FRAP1*, *CD101*, *B2M*, and *VAV3*.

Locus	**Multilocus test *P *value/ Single-locus TDT *P *value**		**Case-control**	**Combined test *P *value**
			
	**Family set 1**	**Family set 1 + 2**		
*NRAMP1*	-	0.56	0.20	0.68
*4-1BB*	0.71	-	-	-
	0.88	-	-	-
	0.52	-	-	-
	0.35	-	-	-
	0.53	-	-	-
	0.29	-	-	-
	0.95	-	-	-
	0.24	-	-	-
*FRAP1*	0.44	-	-	-
*CD101*	0.68	-	-	-
*B2M*	0.90	-	0.11	0.75
*VAV3*	0.26 (block 1)	-	-	-
	0.80 (block 2)	-	-	-
	0.86 (block 3)	-	-	-

**Table 5:** Association analysis of non-synonymous SNPs. SNPs with allele frequencies above 0.05 and the *NRAMP1 *(GT)_n _microsatellite in up to 1,476 families with at least one affected offspring. N, number; T, number of transmissions; NT, number of untransmitted alleles; %T, percentage transmission of minor allele from heterozygous parents to type 1 diabetes offspring (obtained by transmission/disequilibrium test (TDT)); GTRR, genotype relative risk; *P*, probability value (two-sided).

**Locus**	**Marker ID**	**Amino acid change/ alleles**	**Minor allele frequency**	**N families**	**T**	**NT**	**%T**	***P*_TDT_**	***P*_GTRR_**
*VAV3*	DIL1521	Thr293Ser/T>A	0.27	1 476	834	840	49.64	0.77	0.90
	DIL3809	Pro611Ser/G>A	0.13	1 476	417	429	49.29	0.68	0.84
*CD101*	DIL3794	Asn225Ser/A>G	0.32	652	517	515	49.9	0.95	0.96
*NRAMP1*	(GT)_n_	-	-	1 476	-	-	-	-	0.36

With regards to our association study in humans, intronic and potential regulatory regions were not sequenced in the candidate genes since these cover large genomic regions, which will have to wait for much more extensive polymorphism maps [21]. For example, for *VAV3*, which spans almost 400 kb, less than 10% of the genomic region of *VAV3 *was re-sequenced to identify SNPs. The general importance of intronic and intergenic regulatory sequences as candidates for disease susceptibility is well recognised. Hence, potential unidentified causal variants in introns or flanking regions of the genes may have been missed, and remain a target for future analyses. Despite finding no evidence of association, it remains possible that there exists a common disease variant in one or more of the six candidate genes tested, which either has an effect smaller than would be detected with this study or is in much weaker LD with the tag SNPs than any other SNP known to us [22].

Finally, the possibility of one or more rare disease variants in a locus needs to be considered [23]. The best candidates for rare disease variants in the six genes studied here were thus genotyped in an expanded case-control collection of up to 3,704 type 1 diabetes cases and 3,930 controls: DIL5202/ss23142243 causes a non-conservative change in *NRAMP *(Asp543Asn, MAF = 0.02) and DIL3799/ss23142349 in *CD101 *(Val839Ile; MAF = 0.03). For both SNPs, *P *values above 0.05 were obtained (*P *= 0.19 for DIL5202/ss23142243 and *P *= 0.80 for DIL3799/ss23142349), therefore, making it less likely that these rare variants contribute to susceptibility to type 1 diabetes. Nevertheless, causal variants with MAFs less than 0.01 [24] may well remain undetected in our re-sequencing panels of 32 or 96 case DNAs. However, the re-sequencing of several hundred cases and controls is beyond the scope of the present study in which we have investigated variants with MAF ≥ 0.03.

## Conclusion

Taken together, these data make an association between type 1 diabetes and common variation in coding and untranslated regions of the six functional candidate genes in the investigated human-mouse orthologue regions less likely. Several possibilities may account for this. A gene (or several genes) in an *Idd *interval may account for disease susceptibility in the NOD mouse, but the human orthologous region may lack this susceptibility variant. The scenario, in which candidate genes in the NOD *Idd *interval may not necessarily be harbouring a functional, causal variant in their human orthologue genes, was discussed previously [25]. It is also possible that the selected candidate gene in the *Idd *interval may not be the gene causing susceptibility to disease.

The tag SNP maps described here will be useful for association studies of other diseases. They will be integrated into future SNP maps encompassing the entire orthologous regions and all regulatory sequences and genes encoded within them.

## Methods

### Subjects

All family members were white and of European ancestral origin. The type 1 diabetes families comprised two parents and a least one affected child. The 748 type 1 diabetes families used in 'step 1' were as described previously [26]: 472 UK Warren 1 multiplex and 276 multiplex Human Biological Data Interchange families ascertained in the U.S.A. The case-control DNA set for the tag SNP approach consisted of 1,709 Caucasian type 1 diabetes cases, which were recruited from across Britain in the Juvenile Diabetes Research Foundation/Wellcome Trust funded UK Genetic Resource Investigating Diabetes (GRID) study [27], and 1,829 population-based controls from the 1958 British Birth Cohort (BBC) [28]. The mean age-at-onset of the cases, with almost all under 16 years of age at diagnosis, is 7.5 years (with a standard deviation of 4 years). The 1958 BBC controls are part of an ongoing longitudinal study and the subjects are British citizens born in a particular week in 1958. In order to test association for type 1 diabetes susceptibility and the rare variants in *CD101 *and *NRAMP1*, DIL3799/ss23142349 and DIL5202/ss23142243, a total of 3,704 type 1 diabetes cases and 3,930 controls were used.

For 'step 2' genotyping of *NRAMP1*, the 748 type 1 diabetes families described above were used in addition to 343 multiplex/simplex families from the UK, 159 Norwegian simplex families, 322 Romanian simplex families, and 60 multiplex families from the USA totalling the combined DNA sets to 1,632 type 1 diabetes families, as described previously [26].

### Sequencing

Nested PCR products from DNA from 96 or 32 type 1 diabetes patients were sequenced using an Applied Biosystems (ABI) 3700 capillary sequencer (Foster City, CA), and SNPs identified using the Staden Package [29].

### Genotyping

SNPs were genotyped using the Invader^® ^assay (Third Wave Technologies, Inc. Madison WI) [30] and TaqMan MGB chemistry (ABI) [31]. The *NRAMP1 *microsatellite was genotyped on an ABI3700 sequencer using fluorescent primers as previously described [32]. Full details of primers and probes used for genotyping are available upon request. All genotyping data was double-scored independently.

### Annotation

Annotation of *NRAMP1 *(European Molecular Biology Laboratory [EMBL] accession numbers D50402, D50403, BC041787, L32185, BC033754), *FRAP1 *(UO88966), *4-1BB *(UO3387), *CD101 *(Z33642), *B2M *(BC032589) and *VAV3 *(AF118887, *VAV3*; AF118886, *VAV3β*; AF118887, *VAV3.1*) was performed by importing Ensembl information into a temporary ACeDB database as described in Burren *et al. *[33]. After confirmation of gene structures by BLAST analysis, these were re-extracted in GFF format and submitted to a local Gbrowse database (National Center for Biotechnology Information build 34) (DIL annotations viewable at T1DBase [34].

### Statistical analysis

The program for the selection of tag SNPs [17] and association analysis used here are implemented in the *Stata *statistical system and may be downloaded from our website [35]. All genotyping data were in Hardy-Weinberg equilibrium (*P *> 0.05).

## Authors' contributions

LMM and DJS contributed equally to this work by performing the genetic studies and writing the manuscript. AV, FP, RP, CL, JH, HF, CM, KMH, GC carried out the genetic studies and collated data. JDC performed the statistical analysis and participated in the design of the study. LJS participated in the sequence analysis. NW participated in design and collated data. KSR, CG, C I-T, DAS, DPS and LBP participated in the study design and coordination. JAT, LSW and RCT helped to draft the manuscript. All authors read and approved the final manuscript.

## Supplementary Material

Additional File 2 SNPs, including two deletion/insertion polymorphisms, identified in *NRAMP1*. Novel SNPs are denoted by "ss" numbers and previously published SNPs are denoted by "rs" numbers. Minor allele frequencies are based on the sequencing panel of 32 type 1 diabetes subjects. *R*^2 ^values for non-typed SNPs. UTR, untranslated region. Click here for file

Additional File 5 SNPs identified in *CD101*. Novel SNPs are denoted by "ss" numbers and previously published SNPs are denoted by "rs" numbers. Minor allele frequencies are based on the sequencing panel of 96 type 1 diabetes subjects. *R*^2 ^values for non-typed SNPs. Note that DIL3969 has an allelic *R*^2 ^< 0.80 due to technical difficulties with the assay. UTR, untranslated region. Click here for file

Additional File 7 SNPs, including four in/dels identified in *VAV3*. Novel SNPs are denoted by "ss" numbers and previously published SNPs are denoted by "rs" numbers. Minor allele frequencies are based on the sequencing panel of 96 type 1 diabetes subjects. *R*^2 ^values for non-typed SNPs. Note that DIL6496 and DIL6488 in block 1, and DIL1526 have allelic *R*^2 ^values < 0.80, which was due to technical difficulties with those assays. UTR, untranslated region. Click here for file

Additional File 1 Supporting published references for Table 1. NOD mouse *Idd *loci, the location of their human orthologous regions, and selected functional candidate gene within the *Idd *interval. *Estimated number of genes. Click here for file

Additional File 3 SNPs identified in *4-1BB*. Minor allele frequencies are based on the sequencing panel of 96 type 1 diabetes subjects. Novel SNPs are denoted by "ss" numbers and previously published SNPs are denoted by "rs" numbers. Note that DIL4247/rs6694557 could not be genotyped due to assay technical difficulties. UTR, untranslated region. Click here for file

Additional File 4 SNPs identified in *FRAP1*. Novel SNPs are denoted by "ss" numbers and previously published SNPs are denoted by "rs" numbers. Minor allele frequencies are based on the sequencing panel of 32 type 1 diabetes subjects. *R*^2 ^values for non-typed SNPs. UTR, untranslated region. Click here for file

Additional File 6 SNPs identified in *B2M*. Novel SNPs are denoted by "ss" numbers and previously published SNPs are denoted by "rs" numbers. Minor allele frequencies are based on the sequencing panel of 32 type 1 diabetes subjects. *R*^2 ^values for non-typed SNPs. UTR, untranslated region. Click here for file

Additional File 8 Genotyping counts Numbers of attempted subjects for genotyping and of subjects with genotypes. Click here for file
