# Supplementary material for: Construction and analysis of tag single nucleotide polymorphism maps for six human-mouse orthologous candidate genes in type 1 diabetes
Source: BMC Genet. 2005 Feb 18;6:9. doi: 10.1186/1471-2156-6-9 (PMC551616; doi:10.1186/1471-2156-6-9)
Supplement: Additional File 5 — SNPs identified in CD101. Novel SNPs are denoted by "ss" numbers and previously published SNPs are denoted by "rs" numbers. Minor allele frequencies are based on the sequencing panel of 96 type 1 diabetes subjects. R2 values for non-typed SNPs. Note that DIL3969 has an allelic R2 < 0.80 due to technical difficulties with the assay. UTR, untranslated region. [file 1471-2156-6-9-S5.doc]

Table S5: SNPs identified in *CD101.* Novel SNPs are denoted by “ss” numbers and previously published SNPs are denoted by “rs” numbers. Minor allele frequencies are based on the sequencing panel of 96 type 1 diabetes subjects. *R*^2^ values for non-typed SNPs. Note that DIL3969 has an allelic *R*^2^ < 0.80 due to technical difficulties with the assay. UTR, untranslated region.

| Variant names/ dbSNP | **Map position, NCBI build 34** | **Location** | Minor allele frequency | ***R*^2^** |
| --- | --- | --- | --- | --- |
| DIL3967/ ss23142328 | 116888086 | 5´ | 0.27 | 0.97 |
| DIL3968/ ss23142329 | 116888189 | 5´ | 0.27 | tag SNP |
| DIL3969/ rs2806869 | 116888236 | 5´ | 0.46 | 0.58 |
| DIL3970/ ss23142331 | 116888304 | 5´ | 0.02 | - |
| DIL3971/ ss23142332 | 116888361 | 5´ | 0.01 | - |
| DIL3972/ ss23142333 | 116888362 | 5´ | 0.01 | - |
| DIL3786/ rs7537257 | 116889132 | 5´ | 0.05 | tag SNP |
| DIL3787/ ss23142335 | 116890396 | 5´ | 0.29 | 0.89 |
| DIL3788/ rs3818446 | 116890565 | 5´ | 0.33 | 0.93 |
| DIL3789/ ss23142337 | 116891083 | Intron | 0.24 | 0.93 |
| DIL3790/ ss23142338 | 116898790 | Intron | 0.01 | - |
| DIL3791/ rs7554376 | 116898941 | Exon | 0.04 | tag SNP |
| DIL3792/ ss23142340 | 116900427 | Intron | 0.01 | - |
| DIL3793/ ss23142341 | 116900485 | Intron | 0.01 | - |
| DIL3794/ rs3754112 | 116900802 | Exon (Asn225Ser) | 0.32 | tag SNP |
| DIL3795/ ss23142343 | 116902546 | Exon  (Ala327Thr) | 0.02 | - |
| DIL3796/ rs2249265 | 116906107 | Exon (Val415Met) | 0.03 | tag SNP |
| DIL3797/ ss23142345 | 116906417 | Exon  (Arg518Gln) | 0.03 | - |
| DIL3798/ ss23142346 | 116906439 | Exon (Ser525Arg) | 0.04 | 1.00 |
| DIL3978/ rs3736908 | 116907199 | Exon | 0.24 | 0.92 |
| DIL3979/ rs3736907 | 116907310 | Exon | 0.28 | tag SNP |
| DIL3799/ ss23142349 | 116914598 | Exon  (Val839Ile) | 0.03 | - |
| DIL3800/ ss23142350 | 116923672 | Intron | 0.06 | tag SNP |
| DIL3801/ ss23142351 | 116923691 | Intron | 0.01 | - |
| DIL3802/ ss23142352 | 116923804 | Intron | 0.06 | 0.99 |
| DIL3803/ ss23142353 | 116923897 | Intron | 0.01 | - |
| DIL3973/ ss23142354 | 116925835 | 3´ | 0.05 | 0.99 |
| DIL3974/ ss23142355 | 116925888 | 3´ | 0.05 | 0.99 |
| DIL3975/ ss23142356 | 116926037 | 3´ | 0.05 | 0.99 |
| DIL3976/ ss23142357 | 116926067 | 3´ | 0.01 | - |
| DIL3977/ ss23142358 | 116926393 | 3´ | 0.05 | tag SNP |
