# Supplementary material for: Construction and analysis of tag single nucleotide polymorphism maps for six human-mouse orthologous candidate genes in type 1 diabetes
Source: BMC Genet. 2005 Feb 18;6:9. doi: 10.1186/1471-2156-6-9 (PMC551616; doi:10.1186/1471-2156-6-9)
Supplement: Additional File 7 — SNPs, including four in/dels identified in VAV3. Novel SNPs are denoted by "ss" numbers and previously published SNPs are denoted by "rs" numbers. Minor allele frequencies are based on the sequencing panel of 96 type 1 diabetes subjects. R2 values for non-typed SNPs. Note that DIL6496 and DIL6488 in block 1, and DIL1526 have allelic R2 values < 0.80, which was due to technical difficulties with those assays. UTR, untranslated region. [file 1471-2156-6-9-S7.doc]

Table S7: SNPs, including four in/dels identified in *VAV3*. Novel SNPs are denoted by “ss” numbers and previously published SNPs are denoted by “rs” numbers. Minor allele frequencies are based on the sequencing panel of 96 type 1 diabetes subjects. *R*^2^ values for non-typed SNPs. Note that DIL6496 and DIL6488 in block 1, and DIL1526 have allelic *R*^2^ values < 0.80, which was due to technical difficulties with those assays. UTR, untranslated region.

| **LD Block #** | **Variant name/dbSNP** | **Map position, NCBI build 34** | **Location** | **Minor allele frequency** | ***R*^2^** |
| --- | --- | --- | --- | --- | --- |
| 1 | DIL6496/ rs404634 | 107866175 | 5´ | 0.21 | 0.72 |
| 1 | DIL6495/ rs375302 | 107866046 | 5´ | 0.2 | 0.86 |
| 1 | DIL6494/ ss23142374 | 107865968 | 5´ | 0.05 | - |
| 1 | DIL6493/ rs4366347 | 107865881 | 5´ | 0.07 | 0.83 |
| 1 | DIL6492/ rs2779105 | 107865453 | 5´ | 0.18 | tag SNP |
| 1 | DIL6491/ rs2779106 | 107865386 | 5´ | 0.13 | tag SNP |
| 1 | DIL6486/ ss23142378 | 107865208 | 5´ | 0.02 | - |
| 1 | DIL6487/ ss23142379 | 107865180 | 5´ | 0.07 | tag SNP |
| 1 | DIL6488/ rs484091 | 107865128 | 5´ | 0.25 | 0.78 |
| 1 | DIL6489/ ss23142381 | 107865107 | 5´ | 0.26 | 0.81 |
| 1 | DIL6490/ rs483252 | 107865032 | 5´ | 0.16 | 0.88 |
| 1 | DIL6481/ ss23142383 | 107864838 | 5´ | 0.06 | 0.91 |
| 1 | DIL6482/ ss23142384 | 107864831 | 5´ | 0.06 | 1.00 |
| 1 | DIL6483/ ss23142385 | 107864787 | 5´ | 0.02 | - |
| 1 | DIL6484/ rs4558000 | 107864726 | 5´ | 0.24 | 0.92 |
| 1 | DIL6485/ rs4557999 | 107864693 | 5´ | 0.24 | 0.92 |
| 1 | DIL5677/ rs345266 | 107864139 | 5´ | 0.18 | tag SNP |
| 1 | DIL5676/ ss23142389 | 107864117 | 5´ | 0.06 | 1.00 |
| 1 | DIL5675/ rs345267 | 107864104 | 5´ | 0.18 | tag SNP |
| 1 | DIL5674/ rs3761900 | 107863941 | 5´ | 0.06 | tag SNP |
| 1 | DIL5673/ ss23142392 | 107863814 | 5´ | 0.18 | 0.86 |
| 1 | DIL5678/ ss23142393 | 107863772 | 5´ | 0.01 | - |
| 1 | DIL5672/ rs345268 | 107863670 | 5´ | 0.18 | 0.93 |
| 1 | DIL5679/ ss23142395 | 107861660 | Intron | 0.05 | - |
| 1 | DIL5680/ rs3747946 | 107861595 | Intron | 0.02 | - |
| 2 | DIL6426/ ss23142397 | 107685030 | Intron | 0.06 | tag SNP |
| 2 | DIL3890/ ss23142398 | 107684959 | Intron | 0.38 | tag SNP |
| 2 | DIL3892/ ss23142399 | 107684616 | Intron | 0.01 | - |
| 2 | DIL3891/ ss23142400 | 107684684 | Intron | 0.18 | tag SNP |
| 2 | DIL3893/ ss23142401 | 107684600 | Intron | 0.19 | tag SNP |
| 2 | DIL3894/ ss23142402 | 107684226 | Intron | 0.47 | tag SNP |
| 2 | DIL6422/ ss23142403 | 107684067 | Intron | 0.01 | - |
| 2 | DIL5656/ ss23142404 | 107683455 | Intron | 0.07 | tag SNP |
| 2 | DIL5655/ ss23142405 | 107683270 | Intron | 0.01 | - |
| 2 | DIL6427/ ss23142406 | 107682562 | Intron | 0.01 | - |
| 2 | DIL6423/ ss23142407 | 107682472 | Intron | 0.02 | - |
| 2 | DIL6424/ ss23142408 | 107682014 | Intron | 0.4 | 0.86 |
| 2 | DIL6425/ ss23142409 | 107681941 | Intron | 0.36 | 0.85 |
| 2 | DIL3981/ ss23142410 | 107674978 | Intron | 0.02 | - |
| 2 | DIL1522/ ss23142411 | 107674639 | Intron | 0.02 | - |
| 2 | DIL1523/ ss23142412 | 107674608 | Intron | 0.01 | - |
| 2 | DIL1524/ rs7416884 | 107669930 | Intron | 0.37 | 1.00 |
| 2 | DIL1525/ rs4462178 | 107669811 | Intron | 0.37 | tag SNP |
| 2 | DIL4048/ rs6583048 | 107667932 | Intron | 0.19 | tag SNP |
| 2 | DIL1521/ rs7528153 | 107662401 | Exon (Thr293Ser) | 0.27 | tag SNP |
| 2 | DIL1526/ ss23142417 | 107657846 | Intron | 0.37 | 0.77 |
| 2 | DIL3828/ ss23142418 | 107652582 | Intron | 0.03 | - |
| 2 | DIL1528/ ss23142419 | 107648808 | Intron | 0.09 | tag SNP |
| 2 | DIL1529/ rs7518423 | 107648573 | Intron | 0.21 | 0.95 |
| 2 | DIL1530/ rs7516071 | 107648548 | Intron | 0.20 | tag SNP |
| 2 | DIL1531/ ss23142422 | 107648518 | Intron | 0.01 | - |
| 2 | DIL1532/ rs6672483 | 107646934 | Intron | 0.35 | tag SNP |
| 2 | DIL1533/ ss23142424 | 107646687 | Intron | 0.02 | - |
| 2 | DIL3820/ ss23142425 | 107646369 | Exon (Ser461Phe) | 0.01 | - |
| 2 | DIL3815/ rs2494070 | 107601722 | Intron | 0.40 | tag SNP |
| 3 | DIL3816/ rs4526642 | 107601687 | Intron | 0.21 | tag SNP |
| 3 | DIL3888/ ss23142428 | 107587900 | Intron | 0.38 | tag SNP |
| 3 | DIL3889/ rs7527291 | 107587064 | Intron | 0.16 | tag SNP |
| 3 | DIL3813/ ss23142430 | 107585364 | Intron | 0.12 | 0.81 |
| 3 | DIL3814/ ss23142431 | 107585329 | Intron | 0.02 | - |
| 3 | DIL3809/ ss23142432 | 107539983 | Exon (Pro611Ser) | 0.13 | tag SNP |
| 3 | DIL3810/ ss23142433 | 107539975 | Exon (Gln613His) | 0.13 | 0.88 |
| 3 | DIL3811/ ss23142434 | 107539579 | Intron | 0.02 | - |
| 3 | DIL3812/ ss23142435 | 107539520 | Inton | 0.34 | 0.81 |
| 3 | DIL3823/ rs2296877 | 107507304 | Intron | 0.22 | tag SNP |
| 3 | DIL3822/ rs1328208 | 107507006 | Intron | 0.29 | tag SNP |
| 3 | DIL3821/ rs1571342 | 107500042 | Intron | 0.01 | - |
| 3 | DIL4058/ ss23142439 | 107493858 | Intron | 0.02 | - |
| 3 | DIL4046/ rs3748702 | 107493677 | Intron | 0.33 | 0.81 |
| 3 | DIL4047/ ss23142441 | 107493635 | Exon | 0.07 | tag SNP |
| 3 | DIL3827/ rs7549255 | 107470843 | Intron | 0.15 | tag SNP |
| 3 | DIL3826/ ss23142443 | 107470725 | Intron | 0.05 | tag SNP |
| 3 | DIL3825/ rs3187207 | 107470091 | 3´ UTR | 0.01 | - |
| 3 | DIL3824/ ss23142445 | 107469981 | 3´ UTR | 0.01 | - |
| 3 | DIL3864/ ss23142446 | 107469018 | 3´ UTR | 0.01 | - |
| 3 | DIL3865/ rs8676 | 107468539 | 3´ UTR | 0.39 | tag SNP |
| 3 | DIL3866/ rs8458 | 107468530 | 3´ UTR | 0.40 | 0.96 |
| 3 | DIL5657/ ss23142449 | 107465290 | 3´ | 0.02 | - |
