# Supplementary material for: Construction and analysis of tag single nucleotide polymorphism maps for six human-mouse orthologous candidate genes in type 1 diabetes
Source: BMC Genet. 2005 Feb 18;6:9. doi: 10.1186/1471-2156-6-9 (PMC551616; doi:10.1186/1471-2156-6-9)
Supplement: Additional File 1 — Supporting published references for Table 1. NOD mouse Idd loci, the location of their human orthologous regions, and selected functional candidate gene within the Idd interval. *Estimated number of genes. [file 1471-2156-6-9-S1.doc]

Table S1: Supporting published references for Table 1. NOD mouse *Idd* loci, the location of their human orthologous regions, and selected functional candidate gene within the *Idd* interval. *Estimated number of genes.

| ***Idd*** | **Mouse chromosome** | **Interval size (Mb)** | **Number of genes** | **Functional candidate genes in mouse *Idd* intervals** | **Location of human orthologous region** | **Human orthologue genes** | **Known gene function and previously reported disease associations** |
| --- | --- | --- | --- | --- | --- | --- | --- |
| Idd5.2 | 1 | 1.7 | 47 | Idd5.2: Nramp1/Slc11a1 | 2q35 | *NRAMP1/SLC11A1* | Endosomal/lysosomal acidification and associated with protection from infectious disease (1) and susceptibility to autoimmune disease (2) |
| *Idd9.2* | 4 | 1.1 | 13 | *Idd9.2: Frap1* | 1p32 | *FRAP1* | FKBP12-rapamycin associated protein of mTOR. Candidate tumour suppressor gene, whose function in apoptosis is influenced by allelic variation (3). |
| *Idd9.3* | 4 | 1.2 (13, unpublished) | 13 | *Idd9.3: 4-1bb* | 1p36 | *4-1BB* | Role in enhancing and regulating CD4+, CD8+ T cells and dendritic cells (5-7) |
| *Idd10* | 3 | 0.95 (8) | 7 | *Idd10: Cd101* | 1p12 | *CD101* | Co-stimulatory receptor of T cells (9) |
| *Idd13* | 2 | 6 cM (H3a-Il1 interval) (10) | > 50* | *Idd13: B2m* | 15q21 | *B2M* | Required for antigen presentation by MHC class I molecules and the development of diabtes in NOD mice (10, 11) |
| *Idd18* | 3 | 0.7 (12, unpublished ) | 2 | *Idd18: Vav3* | 1p13-p21 | *VAV3* | Guanine nucleotide exchange factor involved in signaling of T and B cell receptors (13-16) |

1. Shaw MA, Collins A, Peacock CS, Miller EN, Black GF, Sibthorpe D, Lins-Lainson Z, Shaw JJ, Ramos F, Silveira F, Blackwell JM: Evidence that genetic susceptibility to Mycobacterium tuberculosis in a Brazilian population is under oligogenic control: linkage study of the candidate genes NRAMP1 and TNFA. *Tuber Lung Dis* 78:35-45, 1997

2. Shaw MA, Clayton D, Atkinson SE, Williams H, Miller N, Sibthorpe D, Blackwell JM: Linkage of rheumatoid arthritis to the candidate gene NRAMP1 on 2q35. *J Med Genet* 33:672-677, 1996

3. Bliskovsky V, Ramsay ES, Scott J, DuBois W, Shi W, Zhang S, Qian X, Lowy DR, Mock BA: Frap, FKBP12 rapamycin-associated protein, is a candidate gene for the plasmacytoma resistance locus Pctr2 and can act as a tumor suppressor gene. *Proc Natl Acad Sci U S A* 100:14982-14987, 2003

4. Lyons PA, Hancock WW, Denny P, Lord CJ, Hill NJ, Armitage N, Siegmund T, Todd JA, Phillips MS, Hess JF, Chen SL, Fischer PA, Peterson LB, Wicker LS: The NOD Idd9 genetic interval influences the pathogenicity of insulitis and contains molecular variants of Cd30, Tnfr2, and Cd137. *Immunity* 13:107-115, 2000

5. Laderach D, Movassagh M, Johnson A, Mittler RS, Galy A: 4-1BB co-stimulation enhances human CD8(+) T cell priming by augmenting the proliferation and survival of effector CD8(+) T cells. *Int Immunol* 14:1155-1167, 2002

6. Wen T, Bukczynski J, Watts TH: 4-1BB ligand-mediated costimulation of human T cells induces CD4 and CD8 T cell expansion, cytokine production, and the development of cytolytic effector function. *J Immunol* 168:4897-4906, 2002

7. Laderach D, Wesa A, Galy A: 4-1BB-ligand is regulated on human dendritic cells and induces the production of IL-12. *Cell Immunol* 226:37-44, 2003

8. Penha-Goncalves C, Moule C, Smink LJ, Howson J, Gregory S, Rogers J, Lyons PA, Suttie JJ, Lord CJ, Peterson LB, Todd JA, Wicker LS: Identification of a structurally distinct CD101 molecule encoded in the 950-kb Idd10 region of NOD mice. *Diabetes* 52:1551-1556, 2003

9. Russell GJ, Parker CM, Sood A, Mizoguchi E, Ebert EC, Bhan AK, Brenner MB: p126 (CDw101), a costimulatory molecule preferentially expressed on mucosal T lymphocytes. *J Immunol* 157:3366-3374, 1996

10. Serreze DV, Bridgett M, Chapman HD, Chen E, Richard SD, Leiter EH: Subcongenic analysis of the Idd13 locus in NOD/Lt mice: evidence for several susceptibility genes including a possible diabetogenic role for beta 2-microglobulin. *J Immunol* 160:1472-1478, 1998

11. Hamilton-Williams EE, Serreze DV, Charlton B, Johnson EA, Marron MP, Mullbacher A, Slattery RM: Transgenic rescue implicates beta2-microglobulin as a diabetes susceptibility gene in nonobese diabetic (NOD) mice. *Proc Natl Acad Sci U S A* 98:11533-11538, 2001

12. Lyons PA, Armitage N, Lord CJ, Phillips MS, Todd JA, Peterson LB, Wicker LS: Mapping by genetic interaction: high-resolution congenic mapping of the type 1 diabetes loci Idd10 and Idd18 in the NOD mouse. *Diabetes* 50:2633-2637, 2001

13. Movilla N, Bustelo XR: Biological and regulatory properties of Vav-3, a new member of the Vav family of oncoproteins. *Mol Cell Biol* 19:7870-7885, 1999

14. Inabe K, Ishiai M, Scharenberg AM, Freshney N, Downward J, Kurosaki T: Vav3 modulates B cell receptor responses by regulating phosphoinositide 3-kinase activation. *J Exp Med* 195:189-200, 2002

15. Fujikawa K, Miletic AV, Alt FW, Faccio R, Brown T, Hoog J, Fredericks J, Nishi S, Mildiner S, Moores SL, Brugge J, Rosen FS, Swat W: Vav1/2/3-null mice define an essential role for Vav family proteins in lymphocyte development and activation but a differential requirement in MAPK signaling in T and B cells. *J Exp Med* 198:1595-1608, 2003

16. Zakaria S, Gomez TS, Savoy DN, McAdam S, Turner M, Abraham RT, Billadeau DD: Differential Regulation of TCR-mediated Gene Transcription by Vav Family Members. *J Exp Med* 199:429-434, 2004
