# Supplementary material for: Construction and analysis of tag single nucleotide polymorphism maps for six human-mouse orthologous candidate genes in type 1 diabetes
Source: BMC Genet. 2005 Feb 18;6:9. doi: 10.1186/1471-2156-6-9 (PMC551616; doi:10.1186/1471-2156-6-9)
Supplement: Additional File 3 — SNPs identified in 4-1BB. Minor allele frequencies are based on the sequencing panel of 96 type 1 diabetes subjects. Novel SNPs are denoted by "ss" numbers and previously published SNPs are denoted by "rs" numbers. Note that DIL4247/rs6694557 could not be genotyped due to assay technical difficulties. UTR, untranslated region. [file 1471-2156-6-9-S3.doc]

Table S3: SNPs identified in *4-1BB*. Minor allele frequencies are based on the sequencing panel of 96 type 1 diabetes subjects. Novel SNPs are denoted by “ss” numbers and previously published SNPs are denoted by “rs” numbers. Note that DIL4247/rs6694557 could not be genotyped due to assay technical difficulties. UTR, untranslated region.

| **Variant name/dbSNP** | **Map position, NCBI build 34** | **Location** | dbSNP | **Minor allele frequency** |
| --- | --- | --- | --- | --- |
| DIL4279/ ss23142250 | 7712555 | 5´ UTR | ss23142250 | 0.03 |
| DIL4278/ ss23142251 | 7712038 | 5´ UTR | ss23142251 | 0.01 |
| DIL4303/ ss23142252 | 7711396 | 5´ UTR | ss23142252 | 0.02 |
| DIL4302/ ss23142253 | 7711389 | 5´ UTR | ss23142253 | 0.01 |
| DIL4301/ ss23142254 | 7711034 | 5´ UTR | ss23142254 | 0.01 |
| DIL4300/ rs519548 | 7711013 | 5´ UTR | rs519548 | 0.01 |
| DIL4299/ rs519546 | 7711012 | 5´ UTR | rs519546 | 0.02 |
| DIL4298/ ss23142257 | 7710951 | 5´ UTR | ss23142257 | 0.01 |
| DIL4297/ ss23142258 | 7710904 | 5´ UTR | ss23142258 | 0.01 |
| DIL4277/ rs226476 | 7708509 | intron | rs226476 | 0.07 |
| DIL4276/ ss23142260 | 7707954 | intron | ss23142260 | 0.01 |
| DIL4275/ ss23142261 | 7707795 | intron | ss23142261 | 0.02 |
| DIL4569/ rs226478 | 7706180 | intron | rs226478 | 0.27 |
| DIL4274/ ss23142263 | 7704579 | intron | ss23142263 | 0.03 |
| DIL4570/ ss23142264 | 7692362 | intron | ss23142264 | 0.33 |
| DIL4282/ rs161826 | 7689884 | intron | rs161826 | 0.02 |
| DIL4281/ ss23142266 | 7689694 | intron | ss23142266 | 0.01 |
| DIL4280/ ss23142267 | 7689577 | intron | ss23142267 | 0.01 |
| DIL4571/ rs679563 | 7680624 | intron | rs679563 | 0.48 |
| DIL4273/ ss23142269 | 7677834 | intron | ss23142269 | 0.40 |
| DIL4272/ ss23142270 | 7676733 | 3´ UTR | ss23142270 | 0.11 |
| DIL4246/ ss23142271 | 7675134 | 3´ UTR | ss23142271 | 0.01 |
| DIL4247/ rs6694557 | 7674826 | 3´ UTR | rs6694557 | 0.18 |
