# Supplementary material for: Construction and analysis of tag single nucleotide polymorphism maps for six human-mouse orthologous candidate genes in type 1 diabetes
Source: BMC Genet. 2005 Feb 18;6:9. doi: 10.1186/1471-2156-6-9 (PMC551616; doi:10.1186/1471-2156-6-9)
Supplement: Additional File 4 — SNPs identified in FRAP1. Novel SNPs are denoted by "ss" numbers and previously published SNPs are denoted by "rs" numbers. Minor allele frequencies are based on the sequencing panel of 32 type 1 diabetes subjects. R2 values for non-typed SNPs. UTR, untranslated region. [file 1471-2156-6-9-S4.doc]

Table S4: SNPs identified in *FRAP1*. Novel SNPs are denoted by “ss” numbers and previously published SNPs are denoted by “rs” numbers. Minor allele frequencies are based on the sequencing panel of 32 type 1 diabetes subjects. *R*^2^ values for non-typed SNPs. UTR, untranslated region.

| **Variant name/dbSNP** | **Map position, NCBI Build 34** | **Location** | dbSNP | **Minor allele frequency** | ***R*^2^** |
| --- | --- | --- | --- | --- | --- |
| DIL4790/ rs7365214 | 11036468 | 5´ | rs7365214 | 0.27 | tag SNP |
| DIL4789/ rs1074078 | 11036334 | 5´ | rs1074078 | 0.27 | 1.00 |
| DIL4791/ ss23142275 | 11034065 | 5´ | ss23142275 | 0.03 | tag SNP |
| DIL4792/ ss23142276 | 11032789 | 5´ | ss23142276 | 0.02 | - |
| DIL4793/ rs2295080 | 11032174 | 5´ | rs2295080 | 0.17 | 0.87 |
| DIL4794/ rs2295079 | 11032111 | 5´ UTR | rs2295079 | 0.17 | tag SNP |
| DIL4795/ rs4845988 | 11029133 | Intron | rs4845988 | 0.20 | 0.85 |
| DIL4796/ ss23142280 | 11029112 | Intron | ss23142280 | 0.02 | - |
| DIL4797/ rs2092642 | 11026866 | Intron | rs2092642 | 0.20 | 0.91 |
| DIL4799/ ss23142282 | 11017368 | Intron | ss23142282 | 0.02 | - |
| DIL4800/ ss23142283 | 11012699 | Intron | ss23142283 | 0.02 | - |
| DIL4801/ rs2076656 | 11011387 | Intron | rs2076656 | 0.03 | - |
| DIL4802/ ss23142285 | 11011260 | Exon | ss23142285 | 0.19 | tag SNP |
| DIL4803/ rs7524202 | 11007308 | Intron | rs7524202 | 0.19 | 1.00 |
| DIL4804/ ss23142287 | 11003505 | Intron | ss23142287 | 0.17 | 0.93 |
| DIL4806/ ss23142288 | 11003338 | Intron | ss23142288 | 0.18 | 1.00 |
| DIL4805/ ss23142289 | 11003188 | Intron | ss23142289 | 0.02 | - |
| DIL4809/ rs2024625 | 11002299 | Intron | rs2024625 | 0.19 | 1.00 |
| DIL4808/ ss23142291 | 11002212 | Intron | ss23142291 | 0.20 | 0.91 |
| DIL4807/ ss23142292 | 11002196 | Intron | ss23142292 | 0.03 | - |
| DIL4812/ rs1064261 | 10998304 | Exon | rs1064261 | 0.19 | 1.00 |
| DIL4811/ rs4845986 | 10998179 | Intron | rs4845986 | 0.19 | 1.00 |
| DIL4810/ rs4845985 | 10998164 | Intron | rs4845985 | 0.19 | 1.00 |
| DIL4813/ ss23142296 | 10936906 | Intron | ss23142296 | 0.02 | - |
| DIL4815/ ss23142297 | 10926808 | Exon | ss23142297 | 0.02 | - |
| DIL4814/ ss23142298 | 10926552 | Intron | ss23142298 | 0.02 | - |
| DIL4816/ rs2275942 | 10916236 | Intron | rs2275942 | 0.02 | - |
| DIL4817/ rs1057079 | 10914604 | Exon | rs1057079 | 0.20 | 0.91 |
| DIL4820/ ss23142301 | 10902954 | Intron | ss23142301 | 0.02 | - |
| DIL4819/ ss23142302 | 10902952 | Intron | ss23142302 | 0.02 | - |
| DIL4818/ ss23142303 | 10902947 | Intron | ss23142303 | 0.02 | - |
| DIL4822/ ss23142304 | 10900385 | Intron | ss23142304 | 0.02 | - |
| DIL4821/ rs2275527 | 10900192 | Exon | rs2275527 | 0.17 | 0.95 |
| DIL4823/ rs3737611 | 10896443 | Intron | rs3737611 | 0.02 | - |
| DIL4824/ ss23142307 | 10894220 | Exon | ss23142307 | 0.02 | - |
| DIL4827/ ss23142308 | 10891176 | Intron | ss23142308 | 0.02 | - |
| DIL4826/ ss23142309 | 10891003 | Intron | ss23142309 | 0.02 | - |
| DIL4825/ ss23142310 | 10890873 | Exon | ss23142310 | 0.16 | tag SNP |
| DIL4828/ ss23142311 | 10886717 | Intron | ss23142311 | 0.02 | - |
| DIL4830/ ss23142312 | 10883877 | Intron | ss23142312 | 0.02 | - |
| DIL4829/ rs1148478 | 10883613 | Intron | rs1148478 | 0.16 | 0.98 |
| DIL4831/ ss23142314 | 10882749 | Intron | ss23142314 | 0.02 | - |
| DIL4832/ rs2275525 | 10879222 | Intron | rs2275525 | 0.02 | - |
| DIL4835/ ss23142316 | 10876692 | Exon | ss23142316 | 0.02 | - |
| DIL4837/ rs2536 | 10876259 | Exon | rs2536 | 0.02 | - |
| DIL4836/ ss23142318 | 10876087 | 3´ | ss23142318 | 0.02 | - |
| DIL4838/ ss23142319 | 10875619 | 3´ | ss23142319 | 0.25 | tag SNP |
| DIL4844/ ss23142320 | 10875059 | 3´ | ss23142320 | 0.02 | - |
| DIL4843/ ss23142321 | 10874999 | 3´ | ss23142321 | 0.02 | - |
| DIL4842/ ss23142322 | 10874986 | 3´ | ss23142322 | 0.02 | - |
| DIL4841/ ss23142323 | 10874942 | 3´ | ss23142323 | 0.02 | - |
| DIL4840/ ss23142324 | 10874882 | 3´ | ss23142324 | 0.02 | - |
| DIL4839/ ss23142325 | 10874828 | 3´ | ss23142325 | 0.02 | - |
| DIL4845/ ss23142326 | 10874631 | 3´ | ss23142326 | 0.02 | - |
| DIL4846/ ss23142327 | 10873343 | 3´ | ss23142327 | 0.02 | - |
