# Supplementary material for: Construction and analysis of tag single nucleotide polymorphism maps for six human-mouse orthologous candidate genes in type 1 diabetes
Source: BMC Genet. 2005 Feb 18;6:9. doi: 10.1186/1471-2156-6-9 (PMC551616; doi:10.1186/1471-2156-6-9)
Supplement: Additional File 8 — Genotyping counts Numbers of attempted subjects for genotyping and of subjects with genotypes. [file 1471-2156-6-9-S8.doc]

| **Table S8: Genotyping** | **counts** |  |  |  |  |  |
| --- | --- | --- | --- | --- | --- | --- |
| **n = number** |  |  |  |  |  |  |
| **Polymorphism** | **Population** | **n families** | **n families** | **n individuals** | **n individuals** | **Genotyping** |
|  |  | **attempted for** | **achieved** | **attempted for** | **attempted for** | **method** |
|  |  | **genotyping** |  | **genotyping** | **genotyping** |  |
| **NRAMP** |  |  |  |  |  |  |
| DIL5186/rs2276631 | Norwegian | 159 | 159 | 672 | 644 | TaqMan |
|  | Romania | 240 | 240 | 864 | 844 |  |
|  | UK | 606 | 606 | 4637 | 4526 |  |
|  | US | 336 | 336 | 1414 | 1398 |  |
|  | UK case-control |  |  | 3824 | 3711 |  |
| DIL5214/rs2279015 | Norwegian | 137 | 137 | 576 | 532 | TaqMan |
|  | Romania | 240 | 240 | 864 | 846 |  |
|  | UK | 638 | 638 | 3980 | 3843 |  |
|  | US | 336 | 336 | 1414 | 1395 |  |
|  | UK case-control |  |  | 4370 | 3634 |  |
| DIL5203/rs1059823 | Norwegian | 137 | 137 | 576 | 532 | Invader |
|  | Romania | 240 | 240 | 864 | 846 |  |
|  | UK | 606 | 606 | 3885 | 3750 |  |
|  | US | 336 | 336 | 1414 | 1395 |  |
|  | UK case-control |  |  | 4370 | 3634 |  |
| DIL5196/rs1809231 | Norwegian | 159 | 159 | 672 | 643 | TaqMan |
|  | Romania | 240 | 240 | 864 | 849 |  |
|  | UK | 606 | 603 | 2956 | 2848 |  |
|  | US | 336 | 335 | 1414 | 1390 |  |
|  | UK case-control |  |  | 3824 | 3712 |  |
| DIL5202/ss23142243 | Norwegian | 90 | 90 | 384 | 366 | TaqMan |
|  | Romania | 197 | 197 | 767 | 733 |  |
|  | UK | 734 | 733 | 3172 | 3041 |  |
|  | US | 345 | 345 | 1434 | 1404 |  |
|  | UK case-control |  |  | 8014 | 7710 |  |
| (GT)n | Norwegian | 69 | 69 | 278 | 262 | Fluorescently- |
|  | Romania | 240 | 240 | 835 | 804 | labelled primers |
|  | UK | 547 | 544 | 2059 | 1926 |  |
|  | US | 336 | 336 | 1398 | 1383 |  |
| **4-1BB** |  |  |  |  |  |  |
| DIL4277/rs226476 | UK | 472 | 469 | 2428 | 2258 | TaqMan |
|  | US | 320 | 320 | 1620 | 1583 |  |
| DIL4569/rs226478 | UK | 471 | 471 | 1858 | 1821 | TaqMan |
|  | US | 276 | 276 | 1128 | 1115 |  |
| DIL4570/ss23142264 | UK | 471 | 470 | 1843 | 1779 | Invader |
|  | US | 276 | 276 | 1127 | 1032 |  |
| DIL4571/rs679563 | UK | 471 | 470 | 2428 | 2347 | TaqMan |
|  | US | 320 | 320 | 1620 | 1563 |  |
| DIL4273/ss23142269 | UK | 471 | 469 | 1858 | 1783 | Invader |
|  | US | 276 | 276 | 1128 | 1083 |  |
| **B2M** |  |  |  |  |  |  |
| DIL7094/ss23142361 | UK | 471 | 469 | 1858 | 1788 | TaqMan |
|  | US | 276 | 275 | 1128 | 1095 |  |
|  | UK case-control |  |  | 3824 | 3668 |  |
| DIL7085/ss23142362 | UK | 471 | 469 | 1858 | 1805 | TaqMan |
|  | US | 276 | 269 | 1128 | 1060 |  |
|  | UK case-control |  |  | 3824 | 3758 |  |
| DIL7086/rs2255235 | UK | 471 | 469 | 1858 | 1782 | TaqMan |
|  | US |  |  | 1508 | 1458 |  |
|  | UK case-control |  |  | 3824 | 3735 |  |
| DIL7083/ss23142365 | UK | 471 | 469 | 1858 | 1805 | TaqMan |
|  | US | 276 | 276 | 1128 | 1089 |  |
|  | UK case-control |  |  | 3824 | 3770 |  |
| DIL7087/rs1869125 | UK | 471 | 469 | 1858 | 1778 | TaqMan |
|  | US | 276 | 275 | 1128 | 1055 |  |
|  | UK case-control |  |  | 3824 | 3332 |  |
| DIL7088/ss23142369 | UK | 471 | 469 | 1858 | 1797 | TaqMan |
|  | US | 276 | 275 | 1128 | 1108 |  |
|  | UK case-control |  |  | 3824 | 3762 |  |
| DIL7089/rs935885 | UK | 471 | 469 | 1858 | 1774 | TaqMan |
|  | US | 276 | 275 | 1127 | 1110 |  |
|  | UK case-control |  |  | 3824 | 3704 |  |
| DIL7082/ss23142371 | UK | 471 | 469 | 1858 | 1761 | TaqMan |
|  | US | 276 | 275 | 1128 | 1089 |  |
|  | UK case-control |  |  | 3824 | 3751 |  |
| **FRAP1** |  |  |  |  |  |  |
| DIL4790/rs7365214 | UK | 471 | 468 | 1843 | 1784 | Invader |
|  | US | 276 | 276 | 1127 | 1110 |  |
| DIL4791/ss23142275 | UK | 471 | 470 | 1843 | 1798 | Taqman |
|  | US | 276 | 276 | 1127 | 1098 |  |
| DIL4794/rs2295079 | UK | 471 | 465 | 1843 | 1681 | Invader |
|  | US | 276 | 253 | 1127 | 883 |  |
| DIL4802/ss23142285 | UK | 471 | 470 | 1843 | 1782 | Taqman |
|  | US | 276 | 276 | 1127 | 1091 |  |
| DIL4825/ss23142310 | UK | 471 | 470 | 1843 | 1779 | Taqman |
|  | US | 276 | 276 | 1127 | 1058 |  |
| DIL4838/ss23142319 | UK | 471 | 470 | 1843 | 1781 | Taqman |
|  | US | 276 | 276 | 1127 | 1091 |  |
| **VAV3** |  |  |  |  |  |  |
| DIL6492/rs2779105 | UK | 472 | 469 | 1843 | 1762 | Invader |
|  | US | 276 | 276 | 1127 | 1098 |  |
| DIL6491/rs2779106 | UK | 471 | 469 | 1843 | 1744 | TaqMan |
|  | US | 276 | 276 | 1127 | 1098 |  |
| DIL6487/ss23142378 | UK | 471 | 469 | 1843 | 1784 | TaqMan |
|  | US | 276 | 275 | 1127 | 1060 |  |
| DIL5677/rs345266 | UK | 471 | 468 | 1843 | 1728 | TaqMan |
|  | US | 276 | 276 | 1127 | 1063 |  |
| DIL5675/rs345267 | UK | 471 | 468 | 1843 | 1777 | TaqMan |
|  | US | 276 | 276 | 1127 | 1100 |  |
| DIL5674/rs3761900 | UK | 471 | 469 | 1843 | 1759 | TaqMan |
|  | US | 276 | 276 | 1127 | 1111 |  |
| DIL6426/ss23142397 | UK | 471 | 469 | 1843 | 1759 | Invader |
|  | US | 276 | 276 | 1127 | 1111 |  |
| DIL3890/ss23142398 | UK | 471 | 471 | 1843 | 1759 | TaqMan |
|  | US | 276 | 276 | 1127 | 1123 |  |
| DIL3891/ss23142400 | UK | 471 | 471 | 1843 | 1737 | TaqMan |
|  | US | 276 | 276 | 1127 | 1111 |  |
| DIL3893/ss23142401 | UK | 471 | 456 | 1843 | 1565 | TaqMan |
|  | US | 276 | 274 | 1127 | 1015 |  |
| DIL3894/ss23142402 | UK | 471 | 471 | 1843 | 1694 | TaqMan |
|  | US | 276 | 276 | 1127 | 1117 |  |
| DIL5656/ss23142404 | UK | 471 | 471 | 1843 | 1817 | TaqMan |
|  | US | 276 | 276 | 1127 | 1088 |  |
| DIL1525/rs4462178 | UK | 471 | 459 | 1843 | 1595 | TaqMan |
|  | US | 276 | 270 | 1127 | 984 |  |
| DIL4048/rs6583048 | UK | 471 | 469 | 1843 | 1772 | TaqMan |
|  | US | 276 | 276 | 1127 | 1053 |  |
| DIL1521/rs7528153 | Norwegian | 159 | 159 | 672 | 623 | TaqMan |
|  | Romanian | 208 | 176 | 768 | 664 |  |
|  | UK | 814 | 801 | 3064 | 2818 |  |
|  | US | 336 | 336 | 1412 | 1394 |  |
| DIL1528/ss23142418 | UK | 471 | 471 | 1843 | 1774 | Invader |
|  | US | 276 | 276 | 1127 | 1100 |  |
| DIL1530/rs7516071 | UK | 471 | 469 | 1843 | 1651 | Invader |
|  | US | 276 | 276 | 1127 | 1061 |  |
| DIL1532/rs6672483 | UK | 471 | 467 | 1843 | 1730 | TaqMan |
|  | US | 276 | 276 | 1127 | 1097 |  |
| DIL3815/rs2494070 | UK | 471 | 470 | 1843 | 1715 | TaqMan |
|  | US | 276 | 276 | 1127 | 1118 |  |
| DIL3816/rs4526642 | UK | 471 | 470 | 1843 | 1694 | TaqMan |
|  | US | 276 | 276 | 1127 | 1118 |  |
| DIL3888/ss23142428 | UK | 471 | 471 | 1843 | 1762 | TaqMan |
|  | US | 276 | 275 | 1127 | 1095 |  |
| DIL3889/rs7527291 | UK | 471 | 470 | 1843 | 1768 | TaqMan |
|  | US | 276 | 276 | 1127 | 1127 |  |
| DIL3809/ss23142432 | Norwegian | 159 | 159 | 672 | 630 | TaqMan |
|  | Romanian | 322 | 310 | 1132 | 999 |  |
|  | UK | 814 | 813 | 3064 | 2924 |  |
|  | US | 276 | 276 | 1127 | 1117 |  |
| DIL3823/rs2296877 | UK | 471 | 469 | 1843 | 1747 | TaqMan |
|  | US | 276 | 276 | 1127 | 1108 |  |
| DIL3822/rs1328208 | UK | 471 | 471 | 1843 | 1732 | TaqMan |
|  | US | 276 | 275 | 1127 | 1090 |  |
| DIL4047/ss23142441 | UK | 471 | 471 | 1843 | 1756 | TaqMan |
|  | US | 276 | 276 | 1127 | 1100 |  |
| DIL3827/rs7549255 | UK | 471 | 470 | 1843 | 1746 | TaqMan |
|  | US | 276 | 271 | 1127 | 995 |  |
| DIL3826/ss23142443 | UK | 471 | 470 | 1843 | 1739 | TaqMan |
|  | US | 276 | 276 | 1127 | 1053 |  |
| DIL3865/rs8676 | UK | 471 | 471 | 1843 | 1756 | TaqMan |
|  | US | 276 | 276 | 1127 | 1100 |  |
| **CD101** |  |  |  |  |  |  |
| DIL3968/ss23142329 | UK | 471 | 446 | 1843 | 1688 | TaqMan |
|  | US | 276 | 269 | 1127 | 1069 |  |
| DIL3786/rs7537257 | UK | 471 | 461 | 1843 | 1627 | TaqMan |
|  | US | 276 | 276 | 1127 | 1119 |  |
| DIL3791/rs7554376 | UK | 471 | 469 | 1843 | 1691 | TaqMan |
|  | US | 276 | 276 | 1127 | 1115 |  |
| DIL3794/rs3754112 | UK | 376 | 376 | 1843 | 1416 | TaqMan |
|  | US | 276 | 276 | 1127 | 1123 |  |
| DIL3796/rs2249265 | UK | 471 | 470 | 1843 | 1736 | TaqMan |
|  | US | 276 | 276 | 1127 | 1099 |  |
| DIL3979/rs3736907 | UK | 471 | 471 | 1843 | 1756 | TaqMan |
|  | US | 276 | 276 | 1127 | 1077 |  |
| DIL3800/ss23142350 | UK | 471 | 471 | 1843 | 1776 | TaqMan |
|  | US | 276 | 276 | 1127 | 1072 |  |
| DIL3977/ss23142358 | UK | 471 | 447 | 1843 | 1684 | TaqMan |
|  | US | 276 | 274 | 1127 | 1099 |  |
| DIL3799/ss23142349 | UK case-control |  |  | 8014 | 7707 | TaqMan |
